# Ketogenic Diets for Body Weight Loss: A Comparison with Other Diets

**DOI:** 10.3390/nu17060965

**Published:** 2025-03-10

**Authors:** Damian Dyńka, Łukasz Rodzeń, Mateusz Rodzeń, Anna Pacholak-Klimas, Georgia Ede, Shebani Sethi, Dorota Łojko, Karolina Bartoń, Ken Berry, Adam Deptuła, Żaneta Grzywacz, Peter Martin, Jen Unwin, David Unwin

**Affiliations:** 1 Institute of Health Sciences, Faculty of Medical and Health Sciences, University of Siedlce, 08-110 Siedlce, Poland; 2 Rodzen Brothers Foundation, 64-234 Wieleń, Poland; 3 Independent Researcher, 197 Lions Mouth Road, Amesbury, MA 01913, USA; 4 Metabolic Psychiatry, Department of Psychiatry and Behavioral Sciences, Stanford University School of Medicine, Palo Alto, CA 94305, USA; 5 Department of Psychiatry, Poznan University of Medical Science, 60-572 Poznan, Poland; 6 Karolina Bartoń Food Coach, 00-586 Warsaw, Poland; 7 Independent Researcher, Holladay, TN 38341, USA; 8 Faculty of Production Engineering and Logistics, Opole University of Technology, 76 Prószkowska St., 45-758 Opole, Poland; 9 Funmed Clinics, Vastra Hamngatan 13A, 41117 Gothenburg, Sweden; 10 The Collaborative Health Community Foundation, Oxford OX2 9HZ, UK; 11 Faculty of Health Social Care and Medicine, Edge Hill University, Ormskirk L39 4QP, UK

**Keywords:** ketogenic diet, low carb, obesity, body weight, weight loss, insulin resistance, inflammation, glycaemic, hunger, appetite, metabolic psychiatry

## Abstract

With the prevalence of obesity and overweight increasing at an alarming rate, more and more researchers are focused on identifying effective weight loss strategies. The ketogenic diet (KD), used as a treatment in epilepsy management for over 100 years, is additionally gaining popularity as a weight loss method. Although its efficacy in weight loss is well documented, the areas where it may be beneficial to other dietary approaches need to be carefully examined. The objective of this paper is to identify the potential benefits of the KD over alternative dietary weight loss strategies based on a comprehensive literature review. It has been shown that the KD may be more bioenergetically efficient than other dietary strategies, inter alia owing to its effect on curtailing hunger, improving satiety and decreasing appetite (influence on hunger and satiety hormones and the sensation of hunger), inducing faster initial weight loss (associated with lower glycogen levels and reduced water retention), and controlling glycaemia and insulinemia (directly attributable to the low-carbohydrate nature of KD and indirectly to the other areas described). These effects are accompanied by improved insulin sensitivity, reduced inflammation (through ketone bodies and avoidance of pro-inflammatory sugars), reduced need for pharmacological obesity control (the diet’s mechanisms are similar to those of medication but without the side effects), and positive impacts on psychological factors and food addiction. Based on the authors’ review of the latest research, it is reasonable to conclude that, due to these many additional health benefits, the KD may be advantageous to other diet-based weight loss strategies. This important hypothesis deserves further exploration, which could be achieved by including outcome measures other than weight loss in future clinical trials, especially when comparing different diets of equal caloric value.

## 1. Introduction

In the wake of its growing prevalence over the past decades, obesity has become one of the major health challenges worldwide. Paradoxically, it is also one of the most neglected public health issues [1,2]. Another paradox is that the problem persists despite the existence of seemingly ready-made solutions to the problem, such as the “Dietary Guidelines for Americans, 2020–2025” [3] and the “National food-based dietary guidelines (FBDGs)” developed for and implemented in more than 100 countries, in association with the Food and Agriculture Organisation of the United Nations (FAO) [4,5]. Hence, one could reasonably expect that people who follow these recommendations would not develop overweight or obesity. Meanwhile, the problem of obesity continues to grow, suggesting that the recommendations are not widely followed. Therefore, clinicians and patients are increasingly turning to other dietary strategies to try to lose weight, including the ketogenic diet.

The ketogenic diet is a high-fat, adequate-protein, low-carbohydrate plan in which total carbohydrate allowance is typically restricted to a maximum of 50 g per day, or 5–10% of daily energy intake. Macronutrient proportions are not strictly prescribed, although protein and fat usually represent 10–30% and 60–80% of daily energy intake, respectively [6,7]. Depending on the goal to be achieved, these proportions will vary. For instance, the classic version of the ketogenic diet (in use since 1921 for epilepsy) contains even more fat and less carbohydrate and protein [8,9]. The aim of a ketogenic diet is always to shift the body into a state of ketosis, in which the main sources of energy are fatty acids and ketones (from consumed fat or mobilised body fat stores) rather than glucose from consumed carbohydrates [10]. The low amount of carbohydrates in a KD promotes lower blood insulin, which is associated with more efficient burning of body fat and increased ketogenesis [11], while high insulin levels will prevent the optimal state of ketosis. In the case of a caloric deficit combined with low carbohydrate intake, the body shifts from using glucose to utilising fatty acids and ketone bodies as primary energy sources (similar to what occurs during fasting). A low intake of glucose leads to its reduced concentration in the serum, which results in decreased insulin secretion, increased glucagon release, and the stimulation of lipolysis (the breakdown of triglycerides into free fatty acids). Free fatty acids undergo beta-oxidation in the liver, producing acetyl-CoA. Due to a shortage of oxaloacetate (resulting from limited glucose metabolism in the Krebs cycle), excess acetyl-CoA is redirected to the ketogenesis pathway in the mitochondria. Through this process, acetyl-CoA is converted into ketone bodies, such as beta-hydroxybutyrate (BHB—the primary ketone body), acetoacetate, and acetone. Consequently, individuals following a ketogenic diet derive their main energy source from ketone bodies rather than glucose [12,13,14,15,16].

Despite its beneficial effects, the ketogenic diet only ranked 25 (out of 30) in the “Best Diets Overall” category in U.S. News & World Report’s 2024 diet comparison. However, in the same comparison, the ketogenic diet ranked first among Best Fast Weight-Loss Diets [17]. However, it is worth noting that the composition of the diet is extremely important. Despite the fact that the state of ketosis can also be achieved by consuming sugar-free carbonated beverages, pepperoni, and other processed low-carbohydrate foods (such as keto sweets), this will not be a well-formulated ketogenic diet and may negatively affect health in the long run. In our opinion, a properly composed ketogenic diet should be based on unprocessed or minimally processed natural ingredients. While some clinicians are also experimenting with exogenous ketones, this paper does not focus on this approach to ketosis.

Our paper aims to assess the efficacy of the ketogenic diet as a weight loss strategy compared to higher carbohydrate diets, while also examining the potential benefits of the ketogenic diet beyond weight loss. Specifically, we explore its impact on hunger regulation, glycaemic control, insulin sensitivity, inflammation, psychological well-being, and the reduced need for obesity medications.

## 2. Methodology

The literature was searched in the PubMed and Google databases using keywords related to the ketogenic diet (KD), weight loss, and metabolic parameters. Selection was based on article titles, abstracts, and full texts. Priority was given to studies from the last 10 years. Section 6.2 discusses only randomised controlled trials (RCTs) (found in the PubMed database) comparing the ketogenic diet with other strategies that prescribe similar energy amounts and/or allow ad libitum consumption.

## 3. Obesity and Overweight

### 3.1. Obesity and Overweight Statistics

For the first time in history, the global number of obese people has exceeded one billion, according to a large-scale study published in *The Lancet* in 2024. It turns out that obesity is currently a greater threat to global health than hunger. In 2022, excess body weight (overweight and obesity) affected approximately 2.5 billion adults, with as many as 890 million suffering from obesity (relative to 1990 figures, the number of obese people grew by 684 million). The largest numbers of obese adults were in the USA, China, and India (2022 data). In the age group 5 to 19 years old, 390 million children were overweight, 160 million of whom suffered from obesity. The problem also affects children under 5 years of age, 37 million of whom were overweight [18,19]. Paradoxically, despite the fact that obesity is currently a more common problem than hunger, obese/overweight people are also often characterised as having coexisting malnutrition [20]. This double burden can be caused by poor-quality foods that contain high energy density yet low nutrient density. A situation then arises in which a person, despite taking in excess dietary energy, is unable to obtain sufficient nutrients, so they are “overfed but undernourished” [21,22].

The statistics and projections are alarming from an economic perspective. Based on 2023 data, the global costs of overweight and obesity are projected to reach USD 4.32 trillion annually by 2035, or almost 3% of the global gross domestic product (GDP) [23]. Going further, another paper that estimated the current and projected costs of overweight and obesity for 161 countries found that by 2060 (relative to 2019), the economic cost of overweight and obesity will increase 15- to 25-fold in low- and middle-income countries, and 4-fold in high-income countries, which will increase obesity-associated costs from 2.19% (in 2019) to 3.29% (in 2060) of the global GDP [24].

### 3.2. Obesity and Overweight Classification

Obesity and overweight involve excessive accumulation of body fat. In a commonly applied classification, overweight in adults is diagnosed at a body mass index (BMI) of 25 to 29.9, while obesity is diagnosed at ≥30; extreme obesity (class III obesity) is defined as BMI ≥ 40 [25,26]. Importantly, however, BMI is an imperfect measure of body fat content (and therefore cannot replace clinical assessment), as it is affected by a number of factors, such as age, gender, and physical activity (and muscle content), although it works fairly well as a screening test [27,28]. Examples of the BMI’s limitations also include its failure to account for differences between muscle and fat mass. This makes it possible for athletes to be classified as overweight despite low levels of body fat. In addition, BMI does not take into account the distribution of body fat (visceral fat is known to be associated with a higher risk of metabolic diseases by increasing the risk of diabetes or myocardial infarction, among others, even in normal-weight individuals) [29]. Given the limitations of BMI alone, there are alternative indicators. For example, waist circumference is a helpful measurement [30], as are waist-to-hip ratio (WHR) and waist-to-height ratio (WHtR). In addition to these indicators, measurements of body fat and lean body mass alone are extremely helpful in assessing overweight and obesity [27,30,31,32]. In children, growth charts are most often used [33].

### 3.3. Obesity—A Disease or a Metabolic Disorder?

Obesity is officially recognised as a disease by many international and national organisations. These include the World Health Organisation, according to which obesity is a complex, chronic disease characterised by excessive fat accumulation [19], and the American Medical Association (AMA) [34], who voted in favour of recognising obesity as a disease in 2013. Many other organisations, such as the American College of Cardiology, the American Association of Clinical Endocrinologists, the Endocrine Society, the American Heart Association, and the American College of Surgeons, also support this idea. Other organisations recognising obesity as a disease [35] include the Polish Society for the Treatment of Obesity (PTLO) [36] and the European Commission [37]. The corresponding code in the 10th International Classification of Diseases is “E66 Overweight and obesity” [38].

However, it should be made clear that defining obesity as a disease and the possible consequences of doing so cause much controversy. Therefore, some organisations and countries do not recognise it as a disease. One of these countries is the UK [39]. Arguments against defining obesity as a disease include the very definition of the term “disease” (the criteria for which obesity does not fully meet, for instance, because it is diagnosed with reference to BMI) [40,41,42], BMI not being a valid indicator for assessing obesity [27], the stigmatisation of obesity as a problem in itself [43] (which may divert obese people’s attention from the true causes of obesity), and—first and foremost—the complexity of the condition.

### 3.4. Causes of Obesity

Although obesity is the result of excessive energy intake, it is necessary to go a step further and try to understand what causes excessive intake in the first place. One possible answer is, of course, hunger, while another relates to psychological issues, such as addiction to highly processed foods (products high in energy density and low in nutrients), towards which the global food industry has shifted [44,45,46]. It also seems important to take note of the interplay between insulin resistance and the development of obesity and the way in which the two influence each other, as this is known to be a vicious circle (for details, see Section 5.4). Importantly, the proportion of thin people in the past was much larger than today. Prior to the second half of the 20th century, obesity was a relatively uncommon condition. It was not until 1977 that a marked increase in the prevalence of obesity and diabetes (along with a few other conditions) was observed [47]. This very recent increase in prevalence seems to indicate the cause cannot be purely genetic; rather, it is much more likely to be triggered by changing environmental factors acting on pre-existing vulnerabilities.

### 3.5. Health Consequences of Overweight and Obesity

The health consequences of excess body fat accumulation are multifaceted and often severe, with risk growing in line with body weight [48]. Excess body weight is known to increase the risk of developing a range of cardiovascular diseases (CVDs), both directly (e.g., by affecting endothelial function) and indirectly (by exacerbating CVD risk factors (e.g., insulin resistance, hypertension, dyslipidaemia, and other metabolic or endocrine changes)) [49,50]. It is also known that obesity significantly promotes the development of type 2 diabetes mellitus (T2DM), as the two conditions are linked in terms of pathophysiology and molecular mechanisms. It is even predicted that the upward trend in obesity is likely to contribute to a type 2 diabetes epidemic in the coming years [51]. Importantly, obesity can also be one of the factors contributing to the development of cancer. It is likely that obesity is the second most important modifiable cause of cancer after smoking [52]. The types most commonly associated with obesity include colorectal, breast, oesophageal, kidney, liver, pancreatic, bladder, and uterine cancers. Additionally, excess body fat increases the risk of cancer mortality by approximately 17% [53]. Overweight and obesity are also associated with a number of other negative health consequences, including increased mortality from any cause, psychiatric disorders, lower quality of life, sleep apnoea, osteoarthritis, and many others [54].

## 4. Ketogenic Diets as a Body Weight Loss Strategy

In addition to its official medical indication for the treatment of epilepsy [9], the ketogenic diet is very often used for weight loss. This is confirmed by, among other things, a study assessing women’s motivations to follow this particular diet. The study found that as many as 77% of the respondents chose the ketogenic diet to lose weight and improve body composition. Interestingly, most women declared that they had achieved their goals [55]. Even among medical students who are not on the ketogenic diet, the majority believe that weight loss is the main reason why people choose to follow it [56]. Other studies also highlight that weight loss is an important reason why people choose a KD [57,58]. Therefore, it is hardly surprising that several meta-analyses and systematic reviews looking at the effect of KD on weight loss have already been published [59,60,61]. While the effect of the ketogenic diet on weight loss is scientifically documented, it is also worth exploring the underlying reasons for its efficacy and considering other areas (if any) in which the KD may be superior to other weight loss approaches.

## 5. Areas Where the Ketogenic Diet May Be More Efficient for Weight Loss than Diets Richer in Carbohydrates

### 5.1. Appetite Regulation

#### 5.1.1. Appetite and Hunger During Weight Loss in a Standard Approach

Standard weight loss diets focus on calorie restriction, typically recommending that the daily energy allowance be 500 to 1000 kcal lower than the body’s requirements [62,63]. In addition, increased physical activity is suggested to help achieve this energy deficit [64]. In commonly recommended diets, the caloric deficit does indeed lead to weight loss, but with a concomitant increase in feelings of hunger and, consequently, appetite [65,66,67]. To mitigate these symptoms, strategies to manage hunger and appetite are suggested, such as drinking more water, increasing fibre in the diet, eating more slowly, choosing solid over liquid foods, etc. [68]. This reasoning ignores the fact that such bodily responses to a sustained caloric deficit are complex and may represent the body’s innate drive to restore energy balance [69,70]. The authors of one paper additionally recognise a dangerous narrative that legitimises the consumption of sweetened beverages and unhealthy foods so long as calories are counted, physical activity is increased, and the energy balance is right [71]. An additional complication may be that the balance generated by exercise is offset by non-exercise physical activity (NEPA); that is, with the introduction of physical training, there is a reduction in other daily physical activities. The authors suggest that this compensatory response is probably even more common than a mere increase in energy intake. This mitigates the caloric deficit, thereby making weight loss more difficult [72]. Obviously, this is a much more complicated consideration [73,74], but nevertheless relevant, considering that the average adult does not exercise much on a daily basis.

Hypothetically, the pattern described above may lead to a situation in which an ordinary person (i.e., not an athlete or physical labourer) who wishes to lose weight by maintaining a negative energy balance will feel hungry and exhausted and therefore will start choosing unhealthy products (which, due to their organoleptic properties, are more appealing, especially to a hungry person with a high appetite) [75]. This choice will be justified by the calorie count, so long as it does not exceed daily energy balance goals. The result will be even greater hunger and appetite, as these types of products are not nutrient-dense and still stimulate the appetite [76,77]. It is also likely that further weight loss will become increasingly difficult, as it is easy to exceed the self-imposed calorie limit by consuming such energy-dense and appealing products [78,79,80]. Then, the already exhausted individual may decide to exercise more to generate an additional caloric deficit, which could translate into an even greater reduction in daily activity (the offset mechanism) [72], creating a vicious cycle. It should be explicitly emphasised that this is only a hypothetical cause-and-effect sequence, which warrants thorough investigation.

#### 5.1.2. Appetite and Hunger During Weight Loss on a Ketogenic Diet

Experiences of appetite, hunger, and satiety during weight loss on a ketogenic diet are completely different than on a calorie-restricted diet (described in Section 5.1.1 above). This is because the ketogenic diet provides a significantly greater feeling of satiety and ease of appetite control compared to diets richer in carbohydrates. In this regard, the KD seems to be superior on a number of levels.

Studies show that ketogenic diets significantly suppress feelings of hunger and reduce the secretion of ghrelin (the hunger hormone) despite weight loss. In a meta-analysis evaluating the effect of KD on appetite suppression, the authors explicitly state that the clinical benefit of the ketogenic diet is that it controls appetite despite weight loss [81]. The mechanisms behind this effect are still not clearly understood, but it is possible that one of the main factors is the effect of ketone bodies themselves (as confirmed by the fact that the administration of exogenous ketone bodies showed a similar effect). The authors of one publication indicate that the higher the level of β-Hydroxybutyrate (BHB) (the main ketone body in the blood), the smaller the increase in ghrelin, the lower the feeling of hunger, and the greater the increase in satiety peptides [82]. Importantly, appetite does not increase (despite weight loss) as long as individuals are in a state of ketosis, whereas when carbohydrates are reintroduced (and the body shifts out of the state of ketosis), feelings of hunger and ghrelin levels increase again to levels even higher than baseline [75,83]. An important point is that weight loss on a ketogenic diet can be achieved without counting calories and without feeling hungry, so people on a KD can eat to satiety and reduce weight at the same time. This is because the perceived level of satiety will not encourage overeating. In one study, patients with type 2 diabetes who followed a ketogenic diet without counting calories reported feeling less hungry at week 10 than at the start of the study, with an average weight loss of 7.2%. Interestingly, these same patients simultaneously reduced their glycated haemoglobin levels, and most of them reduced or even eliminated one or more diabetes medications [84]. The ketogenic diet in patients with T2DM is effective even in the long-term, as a 12-month study confirmed. Patients lost an average of 12% of their body weight during this time (mainly up to month 8, followed by weight maintenance over the following months) [85]. Remarkably, such lasting effects were achieved without deliberate energy restriction or calorie counting and without feeling hungry [70].

Although β-Hydroxybutyrate levels correlate with lower ghrelin concentrations and increased concentrations of satiety hormones (glucagon-like peptide 1 or GLP-1 and cholecystokinin or CCK), they may not correlate with the subjective sensation of appetite, as shown in one study [86]. This was confirmed by a randomised controlled trial (among endurance athletes) showing that fasting and post-meal ghrelin concentrations were lower and GLP-1 concentrations were higher on a ketogenic diet compared to high-carbohydrate diets (HCDs) and habitual diets (HDs); interestingly, subjective appetite ratings did not correlate with objective measures of appetite (such as total ghrelin (GHR), glucagon-like peptide-1 (GLP-1), and insulin levels) [87]. However, another study demonstrated such a relationship by showing a negative correlation between BHB concentrations and feelings of hunger and intention to eat [88]. Yet another study investigated how changes in glycaemia and ketosis affected appetite, executive function, and mood in women following two types of ketogenic diets (an ad libitum ketogenic diet and a commercial energy-restricted ketogenic Mediterranean diet) as compared with the Mediterranean diet. Significant negative correlations of BHB levels with appetite and desire to eat and positive correlations with satiety ratings were demonstrated. The authors concluded that ketogenic diets have a greater effect on appetite reduction compared to the Mediterranean diet [89]. The effect of ketosis on factors related to appetite regulation after diet-induced weight loss was also investigated in another study: After 8 weeks of a very low-calorie ketogenic diet, when participants were in a state of ketosis, the increase in ghrelin induced by weight loss was suppressed. Similarly, leptin, amylin, and subjective appetite sensation levels were lower than after refeeding and coming out of ketosis (i.e., after another 2 weeks) [90]. A further finding about the ketogenic diet is that leptin (the satiety hormone) is decreased rather than increased (which would theoretically be more logical, given that the diet gives a sense of greater satiety), and even more so than on low-fat diets [91]. Leptin is a peptide hormone produced mainly in white adipose tissue and is referred to as the satiety hormone due to its effect on appetite reduction [92]. However, obese individuals are characterised by tissue resistance to leptin, as a result of which their bodies produce more leptin [93]. It is known that the brain’s response to leptin is inhibited by inflammation [94] (and in obesity, this occurs chronically [95], which the ketogenic diet controls effectively [96,97]). This reduction in leptin resistance may represent a key mechanism of action of the ketogenic diet. Hence, the body responds to less leptin with a better satiety response as it is more sensitive to the hormone. This allows obese people to eat to satiety while losing weight until they reach a normal body weight level [70]. One study has found that in the long term (12 months), the ketogenic diet did not alter leptin and ghrelin levels in children, adolescents, and adults affected by GLUT1-Deficiency Syndrome (GLUT1-DS) and drug-resistant epilepsy (DRE) [98]. However, a study among children with epilepsy has shown that leptin levels in children on a ketogenic diet were significantly lower (mean 2.71 ± 0.93 ng/mL) compared to children in the control group (mean 6.14 ± 2.07 ng/mL) (valproic acid (VPA) group: mean 5.85 ± 1.79 ng/mL) [99].

It is also worth noting the impact of individual variability and adherence to the ketogenic diet (KD) in the context of appetite regulation. As mentioned above, studies have shown appetite suppression during ketosis, and after losing this state (primarily triggered by an increase in carbohydrate intake), the feeling of hunger (and ghrelin levels) initially rise again [75,83]. In practice, this may mean that deviations from the diet could disrupt this state, resulting in increased hunger. Additionally, gender appears to modulate some appetite changes observed during weight loss, just as in the initial phase of KD during weight loss, and thus, during a calorie deficit, hunger may increase [83]. Of course, psychological (as described in Section 5.7) and behavioural factors significantly influence the regulation of hunger and satiety. For example, several publications discuss the role of emotions in eating habits and the perception of hunger and satiety [100,101,102,103]. However, the comparison of the impact of psychological and behavioural factors on long-term hunger and satiety regulation, and thus on adherence to the ketogenic diet compared to other nutritional models, remains to be explored.

**Figure 1:**
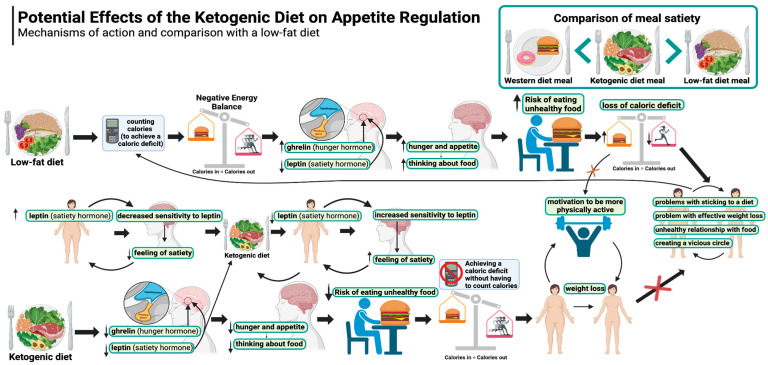
Potential effects of the ketogenic diet on appetite regulation. Created in BioRender. Rodzeń, M. (2025); https://BioRender.com/l21k241 (access date: 8 March 2025).

The potential effects of the ketogenic diet on appetite regulation are illustrated in Figure 1.

### 5.2. Rapid Initial Weight Loss

**Figure 2:**
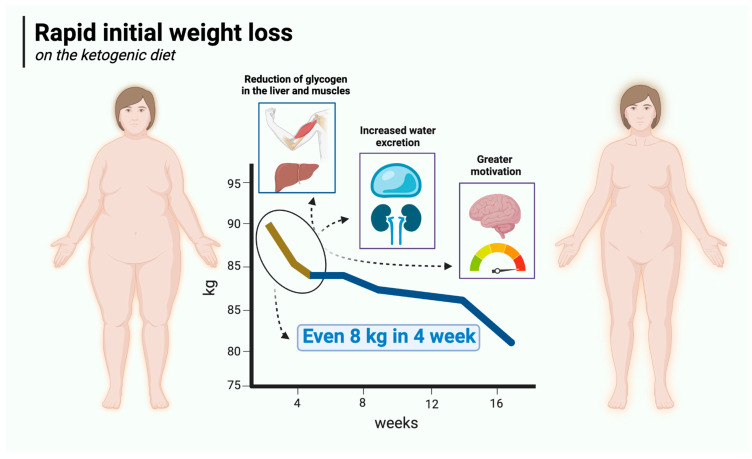
Rapid initial weight loss on the ketogenic diet. Created in BioRender. Rodzeń, M. (2025); https://BioRender.com/l45r024 (access date: 8 March 2025).

Rapid initial weight loss is a major advantage of the ketogenic diet over carbohydrate-based diets. Ketogenic diet users can expect to lose up to 4.5 kg of body weight in the first 2 weeks or even sooner. For the most part, this is due to reduced water retention in the body, which in turn is mainly attributable to low carbohydrate supply, which is intrinsic to this particular diet. Therefore, in its initial stages, the KD has a diuretic effect [104,105,106] similar to that of starvation [107]. It is known that carbohydrate restriction (especially on KD) reduces insulin concentrations [108] (insulin production is mainly stimulated by carbohydrate-rich meals) and depletes glycogen stores [109]. Glycogen is a polymer of glucose that acts as a reserve sugar in animals (corresponding to starch in plants). The main role of glycogen is to ensure glucose homeostasis in the body. To that end, glycogen is regulated by two main hormones—insulin (which promotes its synthesis) and glucagon (which promotes its breakdown) [110]. It is mainly found in the liver (100 g in an average adult) and skeletal muscle (400 g in an average adult), with smaller amounts in the kidneys, heart, and brain. When dietary carbohydrate supply is restricted, glycogen stores in the liver are depleted in just 24 h, and those in the muscles are consumed over the next few days, with a consequent loss of water (bound to glycogen) [109,111,112]. It is generally assumed that 1 g of glycogen is associated with at least 3 g of water (2.7 to 4 g) [113,114,115]. Assuming a 1:3 ratio, a loss of 500 g of glycogen will be accompanied by a loss of approximately 1500 g of water. Thus, the combined effect will be 2 kg of lost body weight. In addition, insulin is known to affect sodium retention in the kidneys, so a reduction in insulin concentration (as observed with the KD) will reduce sodium retention, which in turn leads to increased water excretion by the kidneys [116,117]. The loss of both water and sodium can reduce blood pressure quite rapidly, sometimes resulting in postural hypotension that may necessitate increasing salt in the diet or deprescribing antihypertensive medication [118]. On top of this, the satiating effect of the ketogenic diet described in Section 5.1 makes the initial weight loss significantly greater than in carbohydrate-rich diets. Rapid initial weight loss on the ketogenic diet is illustrated in Figure 2.

### 5.3. Glycaemic Stabilisation

The influence of glycaemia on appetite regulation is extremely important. It is known that hunger and satiety signals are largely influenced by fluctuations in glucose levels, which is particularly evident in the two extremes, which are hypoglycaemia (i.e., glucose values below normal) and hyperglycaemia (glucose values above normal) [119,120,121,122]. It is also known that glucose level fluctuations are mostly caused by carbohydrate-rich meals, which are logically followed by glucose and insulin spikes much greater than those following low-carbohydrate/high-fat meals [123,124]. In fact, before the hormone leptin was discovered, the main known hormone influencing appetite had actually been insulin, which affects glucose levels both directly and indirectly [70,125]. The first papers on the effects of insulin on hunger were written in the first half of the 20th century [126]. In 2021, an extensive study with 1070 participants investigated the correlation between postprandial glucose concentration on the one hand and appetite and subsequent energy intake on the other. Among other things, the authors found that greater postprandial glucose dips were associated with a shorter time to the next meal (which suggests greater hunger) and greater energy intake within 3–4 h after a meal (or even within 24 h) and showed that glucose dips 2–3 h after a meal were a better predictor of subjective feelings of hunger (and subsequent energy intake) than peak (and cumulative) glucose levels within 2 h after a meal. The authors concluded that postprandial glucose level reduction is common and leads to increased hunger and energy intake [127]. This large-scale controlled study was conducted on healthy individuals representing the general population. However, it is also known that in people with type 2 diabetes, carbohydrate restriction, even in 1 meal per day, already significantly reduces postprandial glucose spikes and has a beneficial effect of reducing glycaemia throughout the day, as shown in one randomised controlled trial [128]. Thus, carbohydrate-rich meals, including in particular high-glycaemic-index (GI) meals, lead to a sequence of hormonal and metabolic changes, such as glucose fluctuations. As a result, people experience an increased desire to eat, which in turn leads to excessive energy intake [129,130].

The ketogenic diet, by its very nature, has a very low glycaemic load, as it allows only small amounts of complex carbohydrates and a marginal dose of simple sugars. So, as a diet to control hyperglycaemia, it has obvious advantages. A 2023 randomised controlled trial (RCT) showed that a hypocaloric ketogenic diet was effective in reducing daily glycaemia compared to a low-fat diet, irrespective of weight loss [131]. Another RCT compared the effect of a Mediterranean diet (MD) meal with a KD meal on glucose and insulin levels. It was found that glucose and insulin levels (and insulin release) were significantly lower after the KD meal compared to the MD meal. Mean glucose levels of 85 ± 2 mg/dL peaked to 110 ± 5 mg/dL at 20 min, ending at 78 ± 4 mg/dL after 180 min. Conversely, blood glucose did not increase at any stage after the KD meal, remaining stable at all intervals, reaching 86 ± 2 mg/dL after 180 min. Meanwhile, insulin after the MD meal increased from 40 ± 4 pmol/L to 497 ± 101 pmol/L at 20 min, reaching 190 ± 23 pmol/L after 180 min. After the KD meal, insulin concentrations remained stable (rising from 44 ± 5 pmol/L to 88 ± 12 pmol/L at 30 min and then falling to 48 ± 4 after 180 min) [132]. The superiority of the ketogenic diet over the low-fat diet in terms of glycaemic control has also been demonstrated in a number of meta-analyses. In 2020, one of these meta-analyses concluded that the ketogenic diet, compared to low-fat (carbohydrate-based) diets, is more effective in managing metabolic parameters responsible for controlling glycaemia, body weight, and even lipids in overweight and obese patients—especially those with T2DM [133]. A 2022 meta-analysis found that the very-low-carbohydrate ketogenic diet (VLCK), compared to diets routinely recommended for patients with type 2 diabetes, more effectively reduces glycated haemoglobin at 3 months (by −6.7 mmol/mol (weighted mean difference [WMD])) and at 6 months (by −6.3 mmol/mol (WMD)). In addition, it leads to a much greater weight loss at 3 months (WMD: −2.91 kg) and at 6 months (WMD: −2.84 kg). At 12 months, body weight and HbA1c levels are comparable [134].

**Figure 3:**
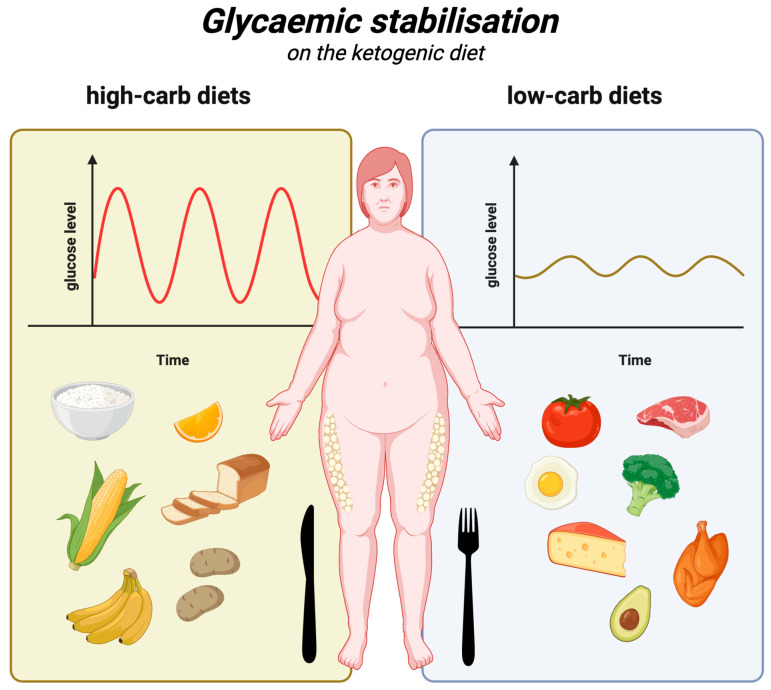
Glycaemic stabilisation on the ketogenic diet. Created in BioRender. Rodzeń, M. (2025); https://BioRender.com/j88s022 (access date: 8 March 2025).

Considering the above, the ketogenic diet, owing to its low-carbohydrate nature, is significantly superior to all diets that are richer in carbohydrates. It is associated with smaller glucose and insulin level fluctuations (compared to high-carbohydrate meals), which in turn prevents the bouts of hunger and overeating commonly associated with such fluctuations. Glycaemic stabilisation on the ketogenic diet is illustrated in Figure 3.

### 5.4. Reducing Insulin Resistance

Insulin resistance is present in up to 4 in 10 American adults and (if assessed using the HOMA-IR index) is associated with increased body weight (based on BMI), body fat, and waist circumference, among other things. In addition, people with insulin resistance are more likely to have low levels of physical activity, hypercholesterolaemia, hypertension, and certain other conditions [135]. The association of obesity with insulin resistance is also known to be linked to inflammatory, neural, and endocrine processes that affect the sensitivity of organs to insulin levels [136]. In obese individuals, a number of factors play a role in developing insulin resistance, including adipose tissue hypoxia, oxidative stress, and endoplasmic reticulum stress; adipose tissue also produces increased amounts of pro-inflammatory cytokines, non-esterified fatty acids, glycerol, and hormones [137]. In view of this, obesity itself may exacerbate insulin resistance, and insulin resistance may also impede weight loss. A growing number of authors refer to the carbohydrate–insulin model (CIM), according to which the weight loss process is about much more than just energy balance. The CIM identifies insulin resistance as a possible root cause of obesity [138,139]. Obviously, this hypothesis does not necessarily exclude the potential importance of a caloric deficit, but it would then be generated spontaneously, without intentional focus on calorie counting. This is well illustrated by studies showing that people on an ad libitum ketogenic diet lose weight anyway (even more than those on higher carbohydrate diets), which is illustrated in more detail in Section 6.2. This is attributable to the satiating effect of KD (described in Section 5.1), owing to which overeating is less common and a negative energy balance is achieved without the need for calorie counting [84,140,141].

**Figure 4:**
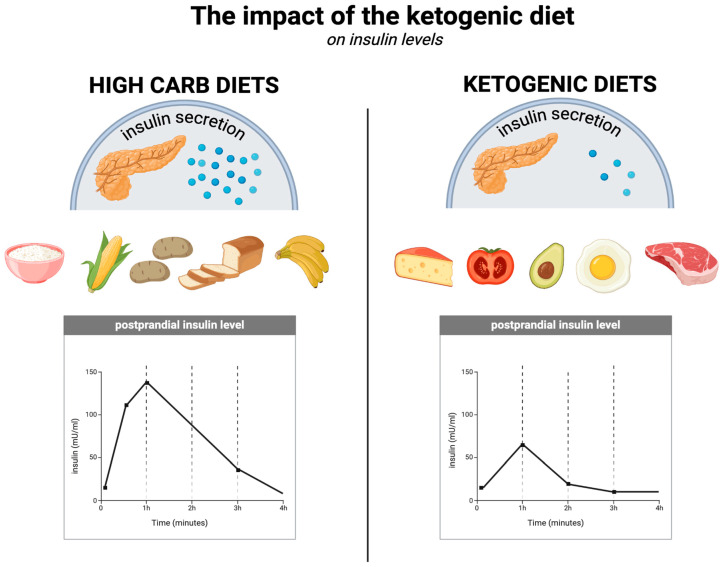
The impact of the ketogenic diet on insulin levels. Created in BioRender. Rodzeń, M. (2025); https://BioRender.com/q23l680 (access date: 8 March 2025).

With respect to insulin levels (which are related to the effects on glycaemia described in Section 5.3) and insulin sensitivity, the ketogenic diet is superior to other diets, as several studies have demonstrated [108]. One randomised controlled trial of overweight or obese individuals with T2DM compared the ketogenic diet without calorie restriction (KD) with a diet recommended by the American Diabetes Association, known as MCCR (medium-carbohydrate, low-fat, calorie-restricted diet). In the KD group, fasting insulin levels decreased significantly more (from an average of 12.2 µIU/mL to 9.3 µIU/mL at 3 months) compared to the MCCR group, in which these levels actually increased (from an average of 10.1 µIU/mL to 11.1 µIU/mL at 3 months). At the same time, the insulin resistance index (HOMA-IR) after 3 months decreased from 1.7 to 1.3 in the KD group and increased from 1.5 to 1.6 in the MCCR group. Additionally, 44% of the KD group members discontinued their diabetes medication, compared to 11% in the MCCR group. In the low-carbohydrate group, participants lost an average of 5.5 kg over this time period, compared to 2.6 kg in the MCCR group, despite not having to restrict calories [142]. It appears that the ketogenic diet can improve insulin sensitivity in just 6 days by reducing fasting insulin concentrations by up to 53%, the insulin resistance index (HOMA-IR) by 57%, and peptide-c by 36% [143]. The KD improves insulin sensitivity in multiple ways, including by maintaining a negative energy balance, reducing fasting insulin concentrations and their diurnal fluctuations (which is attributable to its low-carbohydrate nature), and through direct effects of ketone bodies (among others, on insulin signalling) [11,144]. The impact of the ketogenic diet on insulin levels is illustrated in Figure 4.

### 5.5. Reducing Inflammation

Obesity is strongly associated with chronic (although usually mild to moderate) inflammation, which itself leads to a number of metabolic diseases [145]. Among other things, obesity-induced inflammation is characterised by elevated levels of pro-inflammatory cytokines (e.g., tumour necrosis factor-alpha (TNF-α), interleukin-1 beta (IL)-1β, and IL-6), the polarisation of macrophages towards M1 (pro-inflammatory) from M2 (anti-inflammatory), the activation of Th1 and Th17 cells, and neutrophil influx into adipose tissue [95,146,147].

**Figure 5:**
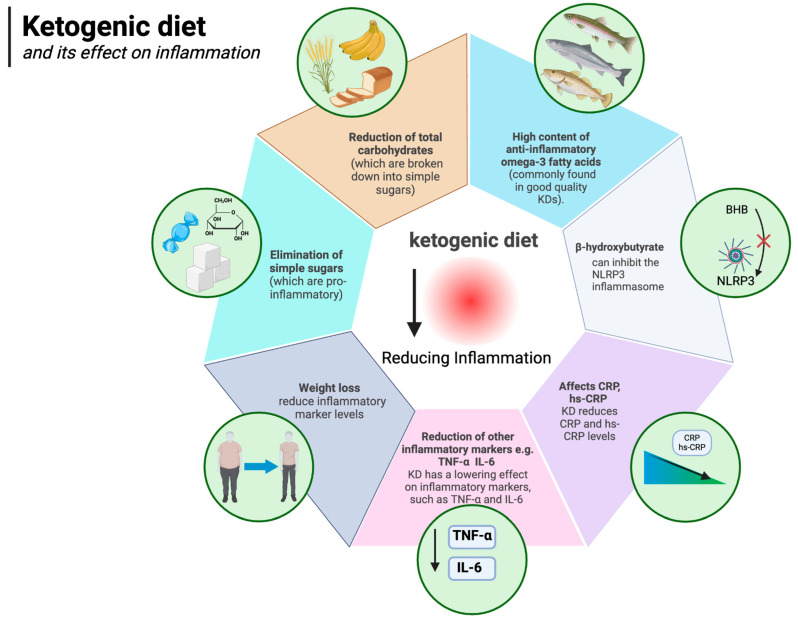
Ketogenic diet and its effect on inflammation. Created in BioRender. Rodzeń, M. (2025); https://BioRender.com/r32o790 (access date: 8 March 2025).

The ketogenic diet is well known to have anti-inflammatory effects. The authors of a 2024 meta-analysis of 44 randomised controlled trials concluded that, compared to other diets, the KD lowers levels of inflammatory markers, such as TNF-α (WMD: −0.32 pg/mL) and IL-6 (WMD: −0.27 pg/mL) (which, as described above, are associated with obesity). It appears that the KD decreases IL-6 levels even more efficiently in obese subjects, BMI > 30 kg/m^2^, compared to those with BMI ≤ 30 kg/m^2^ [96]. It is likely that the anti-inflammatory effect of the ketogenic diet is owed to several factors, including the anti-inflammatory state of ketosis itself, the elimination of simple sugars (which are pro-inflammatory), the elimination of total carbohydrates (which are broken down to simple sugars), and even the high content of anti-inflammatory omega-3 fatty acids (commonly found in good quality KDs) [148,149,150,151,152]. The anti-inflammatory effects of ketosis (which the ketogenic diet can induce) are owed to, among other things, the direct action of β-hydroxybutyrate itself (the main ketone body). Youm et al. showed that BHB can inhibit the NLRP3 inflammasome (which mediates inflammation by being the “centre of command” for pro-inflammatory cytokines) [149]. At the same time, increased expression of NLRP3 inflammasome components is known to be associated with obesity and other inflammatory diseases [153,154,155]. The inflammasome is an important concept in that there is a growing body of research looking at the potentially promising role of NLRP3 inflammasome inhibitor medications in the treatment of inflammatory diseases associated with its activation [156]. The ketogenic diet works in a similar way [157] but without the potential side effects. In addition to the already mentioned reduction in TNF-α and IL-6, a number of studies show that, among other things, the KD reduces CRP and hs-CRP levels [158,159,160]. Furthermore, weight loss alone is known to reduce inflammatory marker levels in the body [161,162,163]. The ketogenic diet, by facilitating weight loss, takes advantage of this important mechanism as well, eventually reducing inflammation. Indeed, it is known that white adipose tissue itself is an important source of obesity-related inflammation [164]. The impact of the ketogenic diet on inflammation is illustrated in Figure 5.

### 5.6. Reduced Need for Obesity Medication

Pharmacological management of obesity is becoming increasingly common. Medications include orlistat, liraglutide, semaglutide, phentermine/topiramate, naltrexone/bupropion and, in certain cases, metreleptin and setmelanotide. Their mechanisms of action largely relate to central effects on increasing satiety, reducing appetite, and at the level of the gastrointestinal tract, delaying gastric emptying [165]. The ketogenic diet can often achieve the same results but arguably with fewer and less serious side effects.

Orlistat inhibits the breakdown and absorption of lipids by inhibiting pancreatic and gastric lipases, i.e., fat-digesting enzymes. It is estimated that orlistat can reduce the absorption of fats by up to 30 per cent (these undigested fats are eliminated with the faeces often causing unpleasant diarrhoea), reducing calorie intake [166] and creating an energy deficit, resulting in weight loss. The ketogenic diet, as demonstrated in a number of studies (described in the previous section), is able to assist in generating an energy deficit without the need to count calories (which is more difficult on carbohydrate-based diets); in this regard it is similar to orlistat, but without the negative side effects [84,140,141].

**Figure 6:**
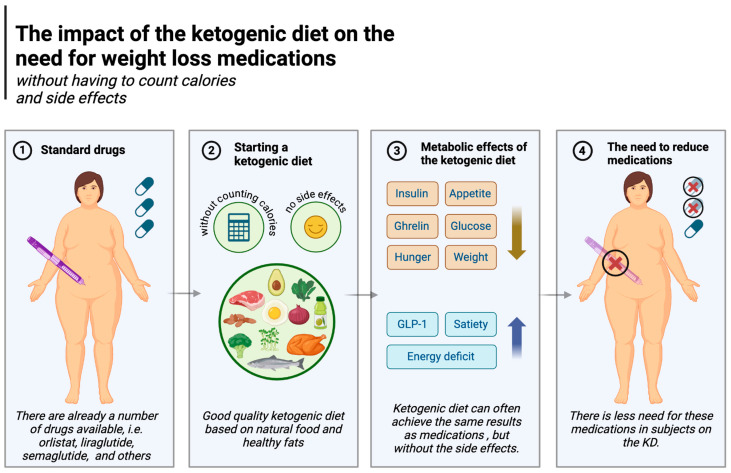
The impact of the ketogenic diet on the need for weight loss medications. Created in BioRender. Rodzeń, M. (2025); https://BioRender.com/z39m516 (access date: 8 March 2025).

Liraglutide and semaglutide are commonly used diabetes drugs also used for weight loss [167,168]. They act as analogues of glucagon-like peptide-1 (GLP-1), a human hormone secreted in the gastrointestinal tract after a meal. Among other effects, the hormone signals the brain by binding to GLP-1 receptors, which reduces hunger and promotes feelings of fullness. It also communicates with the pancreas and stomach, stimulating insulin secretion in response to food intake, leading to a reduction in both post-meal and fasting glucose levels [169,170]. The ketogenic diet also affects these two aspects (reduces hunger and regulates normal glucose levels) and is superior to carbohydrate-based diets in this regard. A randomised controlled trial showed that, compared to carbohydrate-rich diets and habitual diets (HDs), GLP-1 levels were higher in subjects on the ketogenic diet [87]. As explained in Section 5.1, the ketogenic diet is known to induce greater satiety and less appetite. Feelings of hunger do not increase even despite weight loss [75,81,83], and participants in the study by McKenzie et al. (in the T2DM group) not only enjoyed weight loss and less hunger without calorie counting but were also able to discontinue some of their diabetes medication [84]. The KD also reduces glycaemia, another effect of liraglutide and semaglutide. Namely, the diet significantly (and to a much greater degree than other diets) lowers postprandial and fasting glycaemia, as described in detail in Section 5.3. In addition, these results are achieved without any major side effects [108,171]. Other drugs, such as naltrexone/bupropion, or the combination of phentermine and topiramate (especially the former), also reduce appetite (or the sensation of pleasure from eating, as in the case of naltrexone), ultimately lowering energy supply to the body [165,172], which again is similar to the effects of the KD. The impact of the ketogenic diet on the need for weight loss medications is illustrated in Figure 6.

### 5.7. Psychological Advantages

Psychological factors play extremely important roles in weight gain, weight loss, and weight maintenance. Indeed, it is known that psychological factors (like stress) directly affect eating behaviour patterns and adipose accumulation [173,174,175] and are significantly associated with obesity [176]. Maladaptive behaviour patterns of cycling, such as a greater number of past weight loss attempts, weight gain, a greater desire to lose weight, and even weight maintenance, can represent a significant psychological issue, as they have been shown to be associated with an increased risk of depression and anxiety [177]. Treatments to achieve stable eating patterns, such as cognitive behavioural therapy (CBT), are used to reframe negative thoughts and aim to decrease unhealthy eating patterns. In eating disorders, the treatment approach taken must take into consideration these elements. Little is known regarding the role of various dietary patterns in eating disorders.

A growing body of clinical research suggests that the ketogenic diet may offer distinct psychological benefits over other diets [178]. For example, a two-week randomised controlled trial conducted with military personnel compared an isocaloric ketogenic diet with a carbohydrate-based diet, monitoring mood, cognitive function, and subjective sleepiness (during 36 h of extended wakefulness). Compared to the carbohydrate-rich diet, the KD positively improved all these parameters [179].

Hunger is also known to be associated with higher negative and lower positive emotions [100]. As shown in the Minnesota Starvation Experiment, a 6-month period of semi-starvation (1570 kcal per day, a fairly moderate degree of caloric restriction) led to significant changes in the psyche of the male participants. In addition to irritability, apathy, and depression, they dreamed, fantasised, talked, and read about food. Furthermore, they ate slowly and savoured the meals consumed during the period in question, demonstrating the tremendous impact of hunger on psychological well-being [180]. Therefore, it is worth noting that the ability to feel satiated and hunger-free despite being on a diet or undergoing weight loss (as described in detail in Section 5.1) may be an important psychological factor that makes the KD a more enjoyable (and therefore more sustainable) way to lose weight.

While positive psychological effects accompany weight loss regardless of dietary strategy [181,182], promising studies from the emerging field of metabolic psychiatry suggest that the ketogenic diet may offer unique brain health benefits unrelated to weight loss such as reduced neuroinflammation, reduced oxidative stress, and improved brain energy production [183,184,185,186,187].

**Figure 7:**
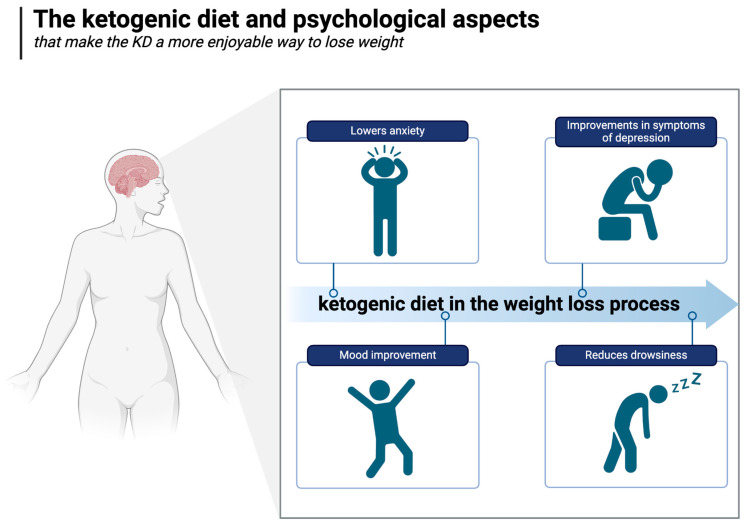
The ketogenic diet and psychological aspects. Created in BioRender. Rodzeń, M. (2025); https://BioRender.com/r80i301 (access date: 8 March 2025).

In an uncontrolled inpatient study of patients with severe, treatment-resistant mental illnesses (major depression, bipolar disorder, and schizoaffective disorder), significant and substantial improvements in symptoms of depression and psychosis were observed within three weeks of adopting a whole-food ketogenic diet, and most patients lost weight despite the fact that most were taking antipsychotic medications known to make weight loss extremely difficult [188]. In the well-known SMILES trial, a 3-month RCT in which it was shown that the Mediterranean diet could improve symptoms of major depression, no significant change in weight was observed [189]. There is also preliminary data indicating the beneficial effect of the ketogenic diet on alleviating or even achieving a complete remission of generalised anxiety [190]. The ketogenic diet and psychological aspects are illustrated in Figure 7.

### 5.8. The KD as a Potential Treatment for Substance Use Disorders and Food Addiction

A number of studies exploring the effect of the ketogenic diet on various forms of addiction have shown promising results. For example, in a study of people with alcohol use disorder (AUD), it has been shown that a state of ketosis induced by the KD helps reduce alcohol cravings, alcohol withdrawal symptoms, and alcohol consumption, leading the study’s authors to conclude that the KD may represent a unique treatment option for AUD [191]. Another study found that in AUD patients, the KD reduces the self-reported need for alcohol and reduces the neurobiological craving signature (NCS) in response to alcohol cues [192]. The KD is now being actively explored by a number of research groups as a potentially therapeutic approach not only for alcohol use disorders [193,194,195] but for other substance use disorders as well [196].

Given the possible similarities in the pathological mechanisms of different addictions, the KD may also be a viable approach to the treatment of addictive eating. It has been postulated that loss of control over food consumption may be driven by ultra-processed foods, particularly products containing refined carbohydrates. Refined and ultra-processed high-GI carbohydrates adversely impact glucose and insulin levels, which may in turn lead to neurochemical response patterns similar to those elicited by chemical addiction [45,197]. By lowering and stabilising glucose and insulin levels, the ketogenic diet may help to reduce food addiction symptoms. Carmen et al. described three cases of obese individuals with coexisting food addiction and binge eating disorder who were placed on a ketogenic diet. These individuals tolerated the KD well, but most importantly, they achieved nearly complete relief from binge eating attacks and substantial improvement in food dependence symptoms (including cravings and lack of control) [198]. In a 2021 study, a very-low-carbohydrate ketogenic diet was shown to be feasible in people diagnosed with food addiction and/or binge eating disorder [199]. Unwin et al. have demonstrated the clinical effectiveness of a short-term, low-carbohydrate group intervention delivered in an online format along with education and social support in people with an addiction to ultra-processed foods [200]. Taking everything into account, the KD appears to hold promise as a therapeutic tool in food addiction and binge eating, although it certainly requires further research and would ideally need to be compared to other dietary strategies.

## 6. Ketogenic Diet and Weight Loss—Overview of Most Recent Studies

### 6.1. Ketogenic Diet and Weight Loss—Background and Results of Meta-Analyses

A number of scientific studies have demonstrated the highly beneficial effects of the ketogenic diet in weight loss, including recent meta-analyses and systematic reviews. A 2024 meta-analysis of 11 randomised controlled trials in obese or overweight women and those with polycystic ovary syndrome (PCOS) showed that compared to controls, the KD significantly reduced body weight (by an average of −9.13 kg), BMI (by an average of −2.93 kg/m^2^), waist circumference (by an average of −7.62 cm), and body fat mass (WMD = −5.32 kg) [59]. Another meta-analysis concluded that the KD could benefit overweight and T2DM patients in terms of weight loss (SMD, −5.63), waist circumference reduction (SMD, −2.32), HbA1c (SMD, −0.38) and triglyceride (SMD, −0.36) reductions, and higher levels of HDL (SMD, 0.28) [60]. Another meta-analysis compared the effects of the ketogenic diet with those of another low-carbohydrate (but not ketogenic) diet in terms of weight and glycaemic control, including in patients with T2DM. Among other things, the KD was shown to significantly improve weight loss and HbA1c levels even more effectively than other low-carbohydrate diets [201]. The greater benefit of a very low-carbohydrate diet compared to diets recommended for those with type 2 diabetes was demonstrated by another meta-analysis in 2022. Weight loss was greater in subjects following very low-carbohydrate diets at 3 months (WMD: −2.91 kg) and 6 months (WMD: −2.84 kg) and was accompanied by lower HbA1c at 3 months and 6 months ([WMD]: −6.7 mmol/mol and −6.3, respectively). At 12 months, the results were comparable [134]. Yet another meta-analysis documented similar benefits at 2 years out [202]. An extensive 2023 meta-analysis of 110 RCTs assessed the effect of carbohydrate restriction on body weight in obese or overweight adults. Each 10% reduction in carbohydrate intake was shown to reduce body weight by an average of 0.64 kg at 6 months and by an average of 1.15 kg at 12 months of follow-up. This linear effect continued for as long as 12 months [203]. There are many more meta-analyses and systematic reviews demonstrating the beneficial effect of the ketogenic diet (and other low-carbohydrate diets) on weight loss [61,144,204,205].

There is a relatively large body of research showing an advantage of the ketogenic diet over other diets. However, the compared diets do not always have an equal caloric value. For example, a 2022 randomised controlled trial compared a 1500–1700 kcal Mediterranean diet with a ketogenic diet below 800 kcal [206]. In a 2020 RCT, a very-low-calorie ketogenic diet (VLCKD) of 600–800 kcal was compared against a standard low-calorie (LC) diet containing 1400–1800 kcal [207]. In view of this, a greater weight loss in the ketogenic group is to be expected on day 1 of the study due to the significant caloric difference. Conversely, a more relevant comparison of the ketogenic diet with other diets (in the context of weight loss) would require studies in which both groups receive an equal number of calories, or at least that both groups consume ad libitum (in which case the intuitiveness of each approach in the context of energy balance can be assessed). The results of such randomised controlled trials from the last 10 years are described below.

### 6.2. Ketogenic Diets and Weight Loss—Randomised Controlled Trials from the Last 10 Years

To ensure a clearer assessment of how the ketogenic diet affects body weight loss and body composition, only randomised controlled trials (RCTs) comparing the ketogenic diet with other strategies that prescribe similar amounts of energy and/or allow ad libitum consumption are discussed in this section. In this way, the caloric differences that could favour either group in terms of weight loss are eliminated. We describe 12 RCTs that meet these criteria.

The first study is a 2024 randomised controlled trial in which the authors compared the ketogenic diet to a control diet (recommended by the Swedish National Food Agency) with a similar calorie count (and physical activity level). The study looked at body composition in healthy women who were neither overweight nor obese. Compared to the control diet, the KD led to greater weight loss—both body fat (reduction in fat mass index (FMI) from 6.2 ± 1.1 to 5.7 ± 1.0 in the KD group vs. from 6.4 ± 0.9 to 6.1 ± 1.0 in the control group) as well as lean mass (reduction in lean mass index (LMI) from 14.8 ± 0.8 to 14.4 ± 0.7 in the KD group vs. from 15.0 ± 0.8 to 14.9 ± 0.8 in the control group) [208]. The loss of lean mass is most likely due to the loss of glycogen and water (the main muscle components), so to make the results more meaningful, a study of this type should be followed by carbohydrate loading (to restore glycogen and water in the muscles). In fact, this was noted by the authors themselves, as they wondered how much of the lean mass loss was due to the loss of muscle mass and how much was due to the loss of glycogen and water.

A second statistically robust RCT conducted in 2021 compared the KD with a Western diet (WD) of similar caloric value (3443.70 kcal ± 545.94 in KD vs. 3529.71 kcal ± 374.06 in WD) in competitive athletes. The authors found that after 2 months, the average body weight in the KD group decreased from 86.39 ± 15.42 kg to 85.51 ± 13.62 kg, as compared to an increase from 89.04 ± 11.73 kg to 90.37 ± 9.91 kg in the WD group (although these differences were not statistically significant). Interestingly, adipose tissue mass decreased more significantly only in the KD group (from 9.86 ± 3.79 kg to 8.42 ± 2.41 kg) compared to a less significant decrease in the WD group (from 10.60 ± 3.92 kg to 9.70 ± 2.53 kg), while fat-free mass increased significantly only in the WD group (from 78.44 ± 8.31 kg to 80.67 vs. from 76.53 ± 12.13 kg to 77.09 in KD). The KD also significantly reduced triglycerides, insulin, glucose, and inflammatory cytokines compared to the Western diet [209].

A third RCT compared the KD with the WD in semi-professional footballers over a 1-month period, with subjects receiving instructions regarding both diets. While total dietary energy intake decreased in both groups (from 2356 ± 450 kcal to 1984 ± 430 kcal in the KD group and from 2146 ± 230 kcal to 1752 ± 320 kcal in the WD group), calories were actually slightly higher in the subjects following the ketogenic diet. Body weight decreased significantly in both groups, but the reduction was slightly greater in the KD group (from 78.19 ± 11.74 kg to 73.98 ± 9.40 kg vs. from 76.15 ± 12.03 kg to 73.76 ± 10.13 kg in the WD group). The KD group also experienced significant reductions in body fat (−1.55 kg vs. −0.92 kg in the WD group), visceral adipose tissue (VAT) (−63 g vs. −27 g in the WD group), waist circumference (−4.19 cm vs. −1.38 cm in the WD group), and extracellular water (-3.43% vs. 0.03% in the WD group). The authors conclude that the KD may be a feasible and safe strategy to lose fat mass in the short term (the study’s duration was only 1 month) and without compromising strength, power, and muscle mass in footballers [210].

A fourth RCT compared the weight loss effect of a Mediterranean ketogenic diet with a calorie-restricted low-fat diet (both models were based on a mobile app). A significant difference was found in favour of the ketogenic diet in terms of weight loss (an average of −5.6 kg vs. −2.5 kg in the low-fat diet group) at week 12. The effect was sustained at week 24 (−8.4 kg in the KD group and −2.9 kg in the low-fat group). Additionally, the KD group experienced greater improvement in glycated haemoglobin levels (reduced by 2.2 mmol/L vs. 0 mmol/L in the low-fat group), liver enzymes (ALT by −15% vs. +7%; AST by −6% vs. +4%), and total bilirubin (−5.6% reduction in the KD group vs. 3.3% increase in the low-fat group) [211].

The fifth RCT compared a hypocaloric ketogenic diet (1200 kcal, 4% carbohydrates, 25% proteins, 71% fats) with a standard low-calorie diet (LCD) (1200 kcal, 40% carbohydrates, 43% proteins, 15% fats) in terms of their effects on weight loss, lean body mass, and resting metabolic rate (RMR) over 4 months. Weight loss was significant in both groups (from an average of 112 kg to an average of 91.8 kg in the KD group and from an average of 106.5 kg to an average of 84.1 kg in the LCD group, without a statistically significant difference between the groups). Body fat mass alone decreased significantly more in the KD group, from an average of 37.5 kg to 21.9 kg vs. from 36.2 kg to 24.2 kg in the LCD group, or by 41.6% vs. 33.1%. In addition, fat-free mass loss was less in the KD group (from 70.5 to 68 kg on average) than in the LCD group (70 to 60 kg). The RMR decrease was also smaller in the KD group (from 2170 kcal/day to 1957.5 kcal/day on average vs. from 2030 kcal/day to 1798 kcal/day in the LCD group) [212].

A sixth RCT compared the effects of four different approaches (all without caloric restriction) on women’s body composition and cardiometabolic health. Three of these were low-carbohydrate (LC) diets (which induced a state of ketosis): one without physical training (as a low-carbohydrate control group: LC-CON), one with high-intensity interval training (LC-HIIT), and one with moderate-intensity continuous training (LC-MICT). The fourth group was a control (CON) without any carbohydrate restrictions. Despite the lack of calorie restrictions, there was significant weight loss in the LC-CON group (average reduction from 65.1 kg to 62.3 kg) as well as in the other low-carbohydrate groups: LC-HIIT (from 67.9 kg to 65 kg on average) and LC-MICT (from 64.5 kg to 61.9 kg on average), as opposed to the CON group (from 66.0 kg to 66.1 kg on average). It is also quite remarkable that even though the women did not have to restrict calories, their caloric intake was nevertheless lower than in the control group (LC-CON vs. CON: 1820 kcal vs. 2074 kcal in week 1; 1680 kcal vs. 2017 kcal in week 2; 1787 kcal vs. 1951 kcal in week 3; and 1790 kcal vs. 2018 kcal in week 4). The additional physical training in the LC-HIIT and LC-MICT groups did not have a significant effect on weight loss (possibly due to the fact that the participants also consumed more calories, thus compensating for the energy lost during training), although it did have an effect on improving cardiorespiratory fitness (CRF) [213].

A seventh RCT compared an ad libitum low-carbohydrate ketogenic diet (LCKD) with an ad libitum usual diet (UD) in competitive weightlifters using a 3-month randomised sequence crossover design. The objective was to assess the diet’s potential as a weight loss strategy in strength sports (powerlifting and Olympic weightlifting). Body weight was significantly lower at the end of the 3-month LCKD phase (−1.7 kg on average, relative to baseline values) compared to that at the end of the 3-month UD phase (+1.56 kg). The mean difference in favour of KD was −3.26 kg. Fat-free mass was also significantly lower after 3 months of LCKD compared to UD (on average −1.74 kg vs. +0.52 kg). The lean body mass loss did not affect the athletes’ strength (their performance did not differ between the two diet phases). The authors of this study note that weight loss was higher than expected based on energy expenditure and calorie intake and after factoring in weight loss associated with glycogen loss. Importantly, energy intake was similar in both phases of the diet [214].

An eighth RCT documented the beneficial effects of an 8-week (ad libitum meal timing and frequency) ketogenic diet on body weight and body composition (compared to the non-ketogenic (ad libitum meal timing and frequency) diet or NDK). Although all participants were allowed to consume an unrestricted amount of calories, only the group on the ketogenic diet experienced weight loss (from an average of 78.8 ± 7.8 kg to 77.4 ± 7.9 kg). Conversely, subjects in the non-ketogenic diet group gained weight, on average, from 74.6 ± 5.3 kg to 75.5 ± 4.9 kg. In addition, compared to the non-ketogenic diet, there was a significant reduction in body fat in the KD group (from an average of 12.0 ± 2.7 kg to 10.9 ± 2.2 kg vs. from an average of 11.3 ± 2.6 kg to 10.9 ± 2.7 kg in NDK) and visceral fat (from an average of 688.9 ± 125.4 g to 592.4 ± 103.1 g vs. from an average of 658.0 ± 200.5 g to 624.2 ± 201.5 g in NDK) [215].

A ninth RCT by Saslow et al. compared the effects of two 32-week online dietary interventions on glycaemic control and other health outcomes in overweight and T2DM patients. One group followed the ketogenic diet without calorie restriction, and the other group (control diet, CD) followed the American Diabetes Association’s (ADA) “Create Your Plate” diet. It turned out that the performance of the ketogenic diet group was significantly superior to that of the control group. Compared to the CD, the KD group experienced significantly greater weight loss relative to baseline values, at both week 16 (−8.5 kg on average (−11.9, −5.2) vs. −3.9 (−8.0, 0.2) in CD) and week 32 (−12.7 kg (−16.1, −9.2) vs. −3.0 (−7.3, 1.3) in CD). Furthermore, HbA1c levels in KD participants decreased more than in the control group (by −0.8 (%) on average (−1.1, −0.6) in KD vs. −0.3 (%) (−0.6, 0.0) in CD), as did triglyceride levels (−60.1 (−91.3, −28.9) in KD vs. −6.2 (−46.0, 33.6) in CD). Importantly, the dropout rate in the KD group was only 8%, while in the control group, it was as high as 46% [140].

A tenth RCT also documented greater weight loss on a low-carbohydrate ketogenic diet (LCK) than a low-fat diet (despite consuming a comparable amount of calories). Subjects in the LCK group did not have to restrict calories, while subjects in the other group (following the moderate-carbohydrate, calorie-restricted, low-fat diet, or MCCR) were additionally instructed to consume 500 kcal less than they actually needed (to trigger weight loss). It turned out that both groups consumed similar amounts of calories anyway, which may indicate a natural, intuitive energy supply adjustment in subjects on the ketogenic diet. Importantly, the LCK group still lost significantly more weight compared to the MCCR group (from an average of 99.9 kg (88.4, 111.5) to 93.8 kg (82.3, 105.3) at month 6 and to 92.0 kg (80.5, 103.6) at month 12, compared to 97.5 kg (86.6, 108.3) to 95.8 kg (84.9, 106.6) at months 6 and 12 in the MCCR group). In addition to weight loss, there was also a greater reduction in HbA1c levels and medication use in LCK subjects over the full 12 months compared to those in the MCCR group [216].

An eleventh RCT compared a very-low-calorie ketogenic diet (VLCKD) with a very-low-calorie diet (VLCD) over 3 weeks. Calorie counts for both diets were 450–500 kcal for women and 650–700 kcal for men. On average, body weight decreased from 99.78 kg (4.57) to 92.80 kg (4.78) in the VLCKD group and from 74.77 kg (5.04) to 68.80 kg (4.24) in the VLCD group. Body fat mass in the VLCKD group fell from 37.24 kg (9.31) to 34.79 kg (9.38), as compared to 33.06 kg (3.60) to 30.59 kg (3.65) in the VLCD group. Conversely, fat-free body mass increased slightly in the VLCKD group (from 53.01 kg (12.86) to 54.93 kg (8.96) on average) and decreased in the VLCD group (from 39.00 kg (3.03) to 35.70 kg (3.09)) [217].

A twelfth RCT compared the effects of two diets on HbA1c levels and other health outcomes in adults with T2DM and obesity or overweight. One group followed a medium-carbohydrate, low-fat, calorie-restricted, carbohydrate-counting diet (MCCR) in line with ADA guidelines, and the other group followed a calorically unrestricted, very-low-carbohydrate ketosis-inducing (ketogenic) diet (LCK). After three months, subjects in the LCK group lost 5.5 kg, while those in the MCCR group only lost 2.6 kg. Remarkably, the LCK subjects did not have to count calories and could eat ad libitum, as opposed to the calorie-restricted MCCR group. In addition, HbA1c levels in the LCK group also decreased (by −0.6 (%) on average) significantly more than in the MCCR group, in which no change from baseline was observed. Importantly, in the MCCR group, only 11% of patients were able to discontinue one or more antidiabetic drugs, as opposed to as many as 44% in the LCK group [142].

**Figure 8:**
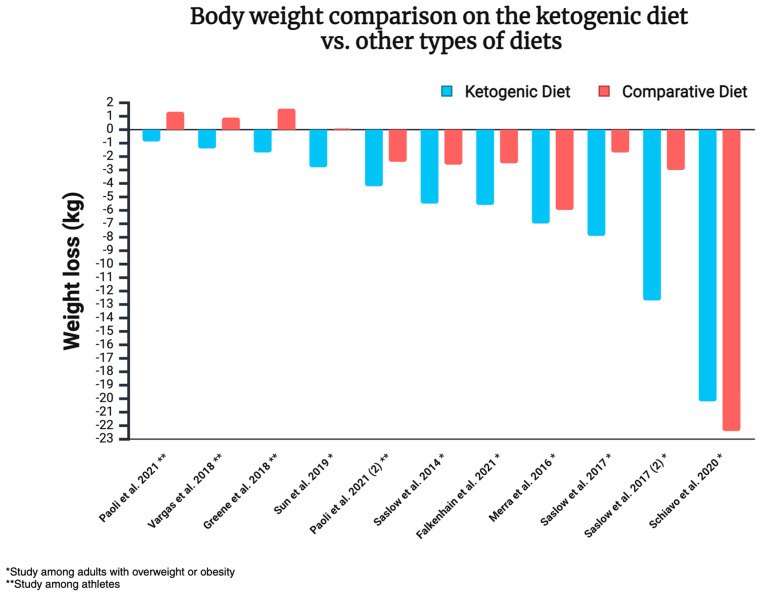
Body weight comparison on the ketogenic diet vs. other types of diets [140,142,209,211,212,213,214,215,217]. Created in https://BioRender.com (access date: 8 March 2025).

The 12 randomised controlled trials discussed here indicate the superiority of ketogenic diets over other dietary approaches in the context of weight loss. The analysed approaches were either equal in terms of calorie supply or allowed ad libitum consumption. In some cases, the energy consumed was actually higher in the KD group (e.g., with a 500 kcal deficit in the control group, without any such restriction imposed on KD subjects). It is worth noting that some of the studies mentioned above focus on individuals with overweight/obesity, while others examine athletes. Therefore, the results of each study cannot be generalised to the entire population. Body weight comparison on the ketogenic diet vs. other types of diets is illustrated in Figure 8.

Some of the RCTs mentioned above involved overweight/obese individuals, while others focused on athletes. The impact of the ketogenic diet on athletic performance still requires thorough investigation, as some studies indicate benefits in terms of fat oxidation, while others show reduced anaerobic performance and muscle glycogen availability, suggesting a lack of optimisation for athletes. One publication stated that KD may not be optimal for improving performance in high-intensity endurance competitions or activities requiring quick bursts of energy powered by carbohydrates [218]. The authors of another publication wrote that in the case of endurance athletes, the literature supports the use of LC/KD as an effective strategy for reducing body weight and fat mass, especially over a period of 3–12 weeks, suggesting a potential improvement in exercise performance at submaximal intensity (~60%) [219]. The complexity of KD’s impact on athletic performance is described, among others, in the document “International Society of Sports Nutrition Position Stand: Ketogenic Diets” [220]. Meanwhile, an interesting 2024 article questions earlier studies suggesting the negative impact of the ketogenic diet on performance, highlighting benefits for athletes adapted to LCHF [221]. Therefore, this topic definitely requires more research.

## 7. The Carnivore Diet as a Type of Ketogenic Diet

The carnivore diet excludes all plant foods and focuses solely on animal foods such as meat, fish, eggs, organs, animal fats, and sometimes dairy products [222]. The lack of plant foods makes the carnivore diet the lowest in carbohydrates of all dietary patterns and therefore often (but not always) naturally lowers insulin levels enough to support ketosis, reducing the body’s reliance on glucose and promoting fat as the primary fuel source. This low-insulin state, combined with the exclusion of certain plant compounds, may offer unique benefits for weight loss. Specifically, the diet omits plant antinutrients (which can interfere with nutrient absorption) like phytates and naturally occurring plant toxins like lectins, which can negatively impact metabolic processes [223]. This section reviews the potential of the carnivore diet to support weight loss, focusing on its potential effects on metabolic efficiency, nutrient density, and gut health relative to plant-based diets.

### 7.1. Current Research on the Carnivore Diet and Weight Loss

Emerging research suggests that the carnivore diet may support weight management, although studies remain limited. A recent survey of over 2000 adherents reported an average BMI reduction from 27.2 to 24.3 after 14 months, with participants noting increased energy and satiety [224]. Similarly, a case study of a 61-year-old woman on a 90% carnivore diet showed a significant weight loss of 32 kg, reducing her BMI from 40 to 28.7 within months [225]. Historical studies, such as those by McClellan and Du Bois, also recorded initial weight reductions among subjects on a meat-only diet [226]. While these findings suggest that carnivore diets can support weight reduction, more rigorous research is needed to assess their safety and efficacy, especially in the longer term.

### 7.2. Potential Mechanisms Underlying Weight Reduction and Metabolic Efficiency

#### 7.2.1. Lectins

Lectins are immune system proteins commonly found in plant foods including grains, legumes, and certain vegetables. They have a unique ability to bind to carbohydrates and can interact with cell receptors, including insulin receptors. Research shows that lectins like wheat germ agglutinin can stimulate these receptors, prolonging signals for fat storage and potentially contributing to insulin resistance [227,228]. By excluding lectin-rich foods, the carnivore diet may support weight management by reducing fat storage signals and improving insulin sensitivity. Lectins can also negatively affect the digestive system (e.g., by disrupting nutrient absorption and interfering with digestive, secretory, or even protective functions of the gastrointestinal tract) or exacerbate inflammation. It is worth noting that soaking or cooking can remove up to 99% of lectins; however, the remaining content is still higher compared to a carnivorous diet. So far, only a limited number of human studies have been conducted in this area, which is why further exploration is needed [229].

#### 7.2.2. Reduced Omega-6 Intake and Inflammation

High omega-6 fatty acid intake, particularly from linoleic acid, has been linked to rising rates of obesity and metabolic disorders [230]. Modern diets now include approximately 29 g of linoleic acid daily compared to less than 2 g in pre-modern diets [231]. Research in mice shows that high linoleic acid consumption can disrupt neurotransmitter signalling, leading to increased food intake and fat accumulation [232]. Diets high in oils such as soybean oil have been shown to be associated with obesity, insulin resistance, diabetes, and fatty liver disease, while canola oil may also contribute to insulin resistance [233,234]. Several studies suggest that reducing the ratio of omega-6 to omega-3 may help protect against chronic diseases by lowering inflammation and supporting metabolic function [235]. By reducing linoleic acid intake, the carnivore diet may improve energy regulation and enhance fat metabolism, which are critical factors for weight loss and metabolic health. Some data suggest that a certain amount of omega-6 fatty acids may potentially reduce cardiovascular risk [236]; however, a high-quality meta-analysis did not confirm this association but did show a positive correlation between serum arachidonic acid (AA) levels and the risk of cardiovascular diseases [237]. Considering the complexity of this issue, including the lean mass hyper-responder phenotype [LMHR], where preliminary data suggest that, under certain conditions (often achievable in a carnivorous diet), even high LDL did not correlate with increased atherosclerosis [238], the balance of potential benefits and risks needs to be determined.

#### 7.2.3. Nutrient Density and Gut Health

A well-formulated carnivore diet is nutrient-dense, providing some nutrients that are virtually impossible to obtain from plant foods such as vitamin B12, as well as more bioavailable forms of key nutrients such as heme iron and the omega-3 fatty acids EPA and DHA from animal foods like beef, liver, and fatty fish [222,223,239]. These nutrients support muscle maintenance, fat metabolism, and energy levels—crucial elements in weight management. Animal products also uniquely supply choline (such as from egg yolks), vitamin K2, and compounds like carnosine and creatine, which may further enhance metabolic health and endurance during weight loss [232]. Calcium deficiency is one possible drawback to the carnivore diet, so attention to calcium supply is suggested [222]. In a 2025 publication, it was rightly noted that it remains to be determined whether the carnivore diet poses a risk of deficiencies in certain trace elements or potentially facilitates a reduced requirement for these nutrients [240]. In contrast, plant-based diets support weight loss through high fibre content and lower calorie density, promoting satiety and stabilising blood sugar [241]. However, plant-based diets too low in or devoid of animal foods lack certain essential nutrients, often requiring supplementation to achieve similar metabolic support [242]. The nutrient density of the carnivore diet may also influence gut health. Animal-based foods lack phytates and other antinutrients, which can affect nutrient absorption and potentially influence immune regulation and the regenerative capacity of intestinal cells [222,235]. Increased bioavailability of minerals in the diet may support gut function and repair processes. Additionally, the absence of fibre and fermentable carbohydrates, which are known to produce gas and may aggravate bloating in sensitive individuals, could reduce gastrointestinal discomfort [214,228]. This decrease in gut fermentation might lower inflammation and reduce the risk of dysbiosis, a microbial imbalance associated with certain metabolic disorders. While fibre generally promotes microbial diversity, some individuals report improved digestive symptoms with reduced fibre intake [243]. However, further research is needed to better understand the short- and long-term effects of carnivore and other dietary patterns on gut microbiota.

### 7.3. Carnivore Diet and Weight Loss—Summary

**Figure 9:**
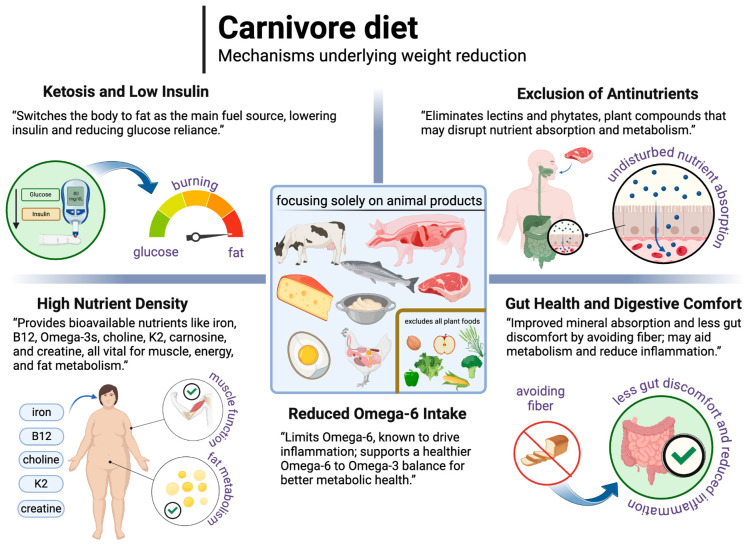
The possible mechanisms underlying the reduction in body weight on the carnivore diet. Created in BioRender. Rodzeń, M. (2025); https://BioRender.com/m25r913 (access date: 8 March 2025).

Well-formulated carnivore diets are generally characterised by low insulin levels, ketosis induction, and reliance on animal-sourced nutrients, which may support weight loss and metabolic health. By excluding plant antinutrients and seed oils high in linoleic acid, carnivore diets could improve both nutrient bioavailability and metabolic function. While preliminary findings suggest potential benefits for weight management, more research is needed to verify longer-term effects and compare outcomes with other types of ketogenic diets. The mechanisms underlying the reduction in body weight on the carnivore diet are illustrated in Figure 9.

## 8. Ketogenic Diet and Health

The beneficial weight loss effects of the ketogenic diet would be questionable if the diet had a detrimental effect on the body. Therefore, this section focuses on the health effects that accompany weight loss in KD subjects.

Based on high-quality scientific evidence provided by meta-analyses and systematic reviews, it is reasonable to assert that the ketogenic diet does not have negative effects on health. In fact (as repeatedly documented above), its effects on health are often positive. For example, a 2022 meta-analysis (of eight RCTs) showed a beneficial effect of the KD on weight loss while also improving such parameters such as HbA1c, triglycerides, and HDL. The authors concluded that a ketogenic diet could be recommended as a therapeutic intervention in overweight and T2DM patients [60]. An earlier meta-analysis demonstrated that by reducing weight in T2DM patients, the KD additionally improves health by lowering fasting glucose, HbA1c, triglycerides, total cholesterol, and LDL cholesterol and by increasing HDL [144]. These benefits are also confirmed by other meta-analyses and systematic reviews mentioned in the earlier sections of this paper [133,134,201].

The ketogenic diet may also help people suffering from a number of different diseases, i.e., diabetes (mainly type 2, but also type 1) [108]. One meta-analysis found that while diets that effectively control glycaemia in T2DM patients include the Mediterranean diet, the low-glycaemic-index (GI) diet, and the moderate carbohydrate diet, the ketogenic diet is nevertheless still the best strategy [244]. The KD may also have beneficial effects on various cardiovascular parameters (e.g., HDL-C, triglycerides, vascular endothelium function, and inflammation) [148,245,246], although some researchers remain concerned about the potential for the KD to increase LDL (and thus total cholesterol) [247]. This particular effect is more likely to occur in normal-weight individuals [248], although the authors indicate that an increase in HDL may compensate for this. Therefore, this issue requires further research and careful monitoring of lipid parameters in people on the ketogenic diet. The KD could also improve patients’ overall mental health (as mentioned earlier) [183,184,185], making it an extremely promising intervention in the field of psychiatry. The KD has also been shown to reverse non-alcoholic fatty liver disease (NAFLD) (through the diet’s effects on a number of mechanisms), as demonstrated in a 2024 paper [249]. It may also help patients suffering from polycystic ovary syndrome (PCOS) [250], lipoedema [251], and certain dermatological disorders [252,253], not to mention over 100 years of safety and efficacy in drug-resistant epilepsy, where it outperforms pharmacotherapy [254,255].

A scientific statement from the National Lipid Association Nutrition and Lifestyle Task Force in 2019 conducted a comprehensive review of the literature on the effects of low-carbohydrate diets (including ketogenic diets) on weight management and other cardiometabolic risk factors. Among the benefits, the authors highlight the positive impact of these diets on triglyceride levels, HDL, glycaemic control, and the potential discontinuation of antidiabetic medications, although after 2 years, no differences are observed in most cardiometabolic markers. The issue of the impact of ketogenic diets on overall cardiovascular risk is frequently raised, citing various (and sometimes conflicting) results regarding LDL levels [256]. It is observed that during ketogenic dieting with concurrent weight loss, LDL levels often decrease (along with a reduction in TG and TC and an increase in HDL) [148]. Conversely, in lean individuals on a ketogenic diet, LDL tends to increase (along with an increase in TC and HDL and a decrease in TG). This occurs in the case of hyper-responders [238]. Clearly, more research is needed in this area to better understand this topic. Additionally, among the areas requiring further exploration is the long-term effect of the ketogenic diet (although it is already a relatively well-studied nutritional model, as it has been used since 1921). The impact of KD on the gut microbiome is also a subject of research, as it is increasingly suggested that the therapeutic benefits of KD in epilepsy treatment may largely result from the modulation of the microbiome by this dietary model. Interestingly, although KD may reduce bacterial diversity, this does not correlate with increased inflammation, but it does coincide with the alleviation of epilepsy symptoms [257,258,259]. Therefore, this topic requires further investigation. It is worth noting, however, that the ketogenic diet should be well balanced to maximise its benefits. One study showed that the standard KD contained lower amounts of magnesium, calcium, iron, phosphorus, and potassium than recommended. Nevertheless, it is noteworthy that the serum concentrations of these elements were always within the reference range, although calcium levels significantly decreased [260]. Therefore, the ketogenic diet, like any other nutritional model, should provide the necessary vitamins and minerals.

It is worth noting that most studies focus on short-term weight loss (from weeks to months), so it is now important to conduct more long-term research. Similarly, the dropout rate from ketogenic diets compared to control diets requires further investigation, as it varies significantly across studies, ranging from 13% to 84% [261]. In a meta-analysis involving a total of 1307 participants on a ketogenic diet and 1294 participants on control diets, the dropout rate for KD was 24.4%, while for control diets, it was 24%, suggesting a similar rate [262]. However, it is worth exploring which factors influence this. In addition, there is still a limited amount of research on metabolic changes related to thyroid function and the impact of KD on T3, T4, or TSH. One RCT showed a significant decrease in T3 concentration after KD intervention [263]. The impact of the ketogenic diet on kidney disease [107], gout [264,265], skin diseases [266], and inflammatory bowel diseases (as preliminary studies suggest promising results [225]) should also be more frequently investigated.

## 9. Contraindications and Side Effects of the Ketogenic Diet

Like any therapeutic tool, the ketogenic diet is not appropriate for everyone; people with certain conditions should not use it. Some absolute contraindications include pyruvate carboxylase deficiency, fatty acid beta oxidation disorders, and primary carnitine deficiencies [267]. Of course, these are just some of the contraindications. Those with serious illnesses affecting major organ systems, such as acute pancreatitis or liver failure, among others, also deserve special attention [268,269]. Given that the ketogenic diet is a potent tool, medical supervision is recommended, especially in patients with coexisting medical or psychiatric conditions and those taking prescription medication. A common example is patients with type 2 diabetes who take hypoglycaemic drugs. Because the ketogenic diet generally works very well to lower blood glucose, the blood glucose must be carefully monitored, and doses of these drugs must be adjusted (reduced) accordingly so as to prevent hypoglycaemia [108]. The same principle applies to other conditions, i.e., patients with hypertension who are taking hypotensive drugs. Because the KD can lower blood pressure, blood pressure and pulse parameters must be monitored, and doses of these drugs may need to be reduced to prevent hypotension [148].

With respect to potential side effects of the ketogenic diet, the vast majority of publications report effects related to the period of adaptation to the ketogenic diet and the so-called keto-flu, i.e., short-term discomforts, which (given the nature of the ketosis state) seems to be a natural reaction of the body to a reduction in glucose and insulin levels. These adaptation symptoms may include headaches, fatigue, lethargy, feeling faint, polyuria, constipation, and nausea [268,269,270,271]. It is also important to make a clear distinction between diabetic ketoacidosis and nutritional ketosis, as these terms are still sometimes confused today. Diabetic ketoacidosis is a condition in which there is simultaneously a significantly increased glucose level (most often >250 mg/dL) and a markedly increased concentration of ketone bodies (15–25 mmol/L, virtually unattainable through nutritional ketosis, as basal insulin production will suffice to suppress uncontrolled ketone production in those with normal pancreatic function) [7,108,272,273,274]. However, this should not be completely ignored, as there is an increased risk of ketoacidosis in people especially with type 1 diabetes, and the introduction of a ketogenic diet in the absence of diabetes control may indeed carry some risk. This is confirmed by the case of a 22-year-old female patient with undiagnosed type 1 diabetes who started KD and developed ketoacidosis [275]. It is worth noting that there are examples of ketoacidosis in which glucose remains normal (such as the ketoacidosis that can occur in people taking SGLT-2 inhibitors and cases of breastfeeding-associated ketoacidosis) [276,277]. This should also be clearly distinguished from the state of nutritional ketosis.

## 10. Limitations

The review was not a systematic review and therefore did not assess all available results. For example, in the review of RCTs, we selected only those studies that did not favour the ketogenic diet and did not impose a greater caloric deficit in the KD groups. In this context, we also consider this to be an advantage of this section. The review included studies involving both individuals with excessive body weight and athletes with high levels of physical activity (especially in the section specifically focused on weight loss). Therefore, not all results from the cited studies can be directly extrapolated to the general population with overweight or obesity.

## 11. Summary

In summary, the ketogenic diet may be superior to other diets in terms of weight loss while maintaining or even improving other health parameters. Some key points are as follows:

1. A ketogenic diet offers an improved regulation of hunger and satiety. It is known that hunger and appetite increase on low-calorie diets, making the weight loss process much more difficult. Conversely, the ketogenic diet increases the feeling of satiety and mitigates the feeling of hunger, even despite a negative energy balance and weight loss, unlike in carbohydrate-based diets.
2. Weight loss is greater during the initial phase of the ketogenic diet, as it reduces water retention and lowers glycogen levels in the body. In addition to the effect on total body weight, this may be important from the psychological perspective, as individuals who experience rapid weight loss will be more motivated to adhere to the diet.
3. The spikes and diurnal fluctuations in glucose and insulin concentrations are smaller because KD meals do not raise glucose levels or insulin levels as much as carbohydrate-based meals (even in those with a low glycaemic index). In the absence of pronounced glucose fluctuations, bouts of hunger and overeating during the day are less likely, and overall daily glycaemia (as demonstrated by HbA1c levels) is improved. Lower glucose levels support lower insulin levels, the sensitisation of body cells to this hormone, and reduced insulin resistance, which often accompanies obesity and overweight and hinders weight loss.
4. Ketogenic diets may help reduce inflammation associated with overweight and obesity to a greater extent than weight loss alone; thus, further research is needed in this area.
5. The effect of the ketogenic diet (unlike carbohydrate-based diets) offers similar potential benefits to those of obesity medications but without the side effects. Thus, there is less need for these medications in subjects adhering to this diet. Obesity drugs reduce energy intake by increasing a sense of satiety, reducing appetite (by acting centrally or reducing glucose fluctuations), and/or inhibiting the digestion of fats. The ketogenic diet reduces the feeling of hunger (e.g., by affecting the hunger and satiety hormones), improves glycaemic control, and also lowers energy intake, even without intentional calorie restriction (which is more difficult to achieve with other dietary approaches).
6. Weight loss on the KD can be more enjoyable (relative to low-fat diets), as it can simultaneously improve mood and cognitive function. The KD may even reduce symptoms of food addiction and binge eating. Many studies show that the beneficial (even therapeutic) psychological effects of ketogenic diets are superior to those of carbohydrate-based diets, exceeding those expected from weight loss alone (although there is a synergy between the two, as described in previous paragraphs).
7. The best results are achieved by using a low-calorie ketogenic diet (and the calorie deficit itself can often be achieved intuitively).

Multiple RCTs indicate that the ketogenic diet is more beneficial in body weight and fat mass loss compared to other dietary interventions, even when caloric intake is equal in the compared groups and/or both groups consume ad libitum. In fact, in some studies, subjects in the group using another diet were asked to reduce their energy intake compared to the ketogenic diet group. An issue requiring a detailed investigation is the loss of lean tissue mass, although the conflicting data (relating to the loss or gain of lean tissue mass) are likely to be caused by the loss of muscle glycogen and water (directly linked to lean tissue mass).

More well-designed studies comparing the ketogenic diet to other approaches are nevertheless necessary to account for any factors that could potentially favour any of the studied interventions (such as imposed differences in energy intake on different diets or varying levels of physical activity).

## 12. Conclusions

The ways in which the ketogenic diet may be superior to other dietary interventions include better regulation of satiety and hunger, greater initial weight loss, favourable effect on glycaemic levels and fluctuations, favourable effect on insulin resistance, reduced inflammation, less need for obesity medication (as the effect of the diet itself is similar, but without the side effects), and positive psychological impact.

The effect of the ketogenic diet on lean body mass is inconclusive, so more well-designed studies are therefore necessary to better understand the effect of the KD on lean body mass.

## Data Availability

Not applicable.
