# Correction: Dyńka et al. Ketogenic Diets for Body Weight Loss: A Comparison with Other Diets. *Nutrients* 2025, *17*, 965

**DOI:** 10.3390/nu18111701

**Published:** 2026-05-27

**Authors:** Damian Dyńka, Łukasz Rodzeń, Mateusz Rodzeń, Anna Pacholak-Klimas, Georgia Ede, Shebani Sethi, Dorota Łojko, Karolina Bartoń, Ken Berry, Adam Deptuła, Żaneta Grzywacz, Peter Martin, Jen Unwin, David Unwin

**Affiliations:** 1 Institute of Health Sciences, Faculty of Medical and Health Sciences, University of Siedlce, 08-110 Siedlce, Poland; 2 Rodzen Brothers Foundation, 64-234 Wieleń, Poland; 3 Independent Researcher, 197 Lions Mouth Road, Amesbury, MA 01913, USA; 4 Metabolic Psychiatry, Department of Psychiatry and Behavioral Sciences, Stanford University School of Medicine, Palo Alto, CA 94305, USA; 5 Department of Psychiatry, Poznan University of Medical Science, 60-572 Poznan, Poland; 6 Karolina Bartoń Food Coach, 00-586 Warsaw, Poland; 7 Independent Researcher, Holladay, TN 38341, USA; 8 Faculty of Production Engineering and Logistics, Opole University of Technology, 76 Prószkowska St., 45-758 Opole, Poland; 9 Funmed Clinics, Vastra Hamngatan 13A, 41117 Gothenburg, Sweden; 10 The Collaborative Health Community Foundation, Oxford OX2 9HZ, UK; 11 Faculty of Health Social Care and Medicine, Edge Hill University, Ormskirk L39 4QP, UK

In the original publication [1], there are errors in the Funding and the Conflicts of Interest statements.

The updated Funding statement appears below:

**Funding:** The publication was financed by the Rodzen Brothers Foundation, founded by two of the co-authors (M.R. and Ł.R.). The funder was involved in the preparation of the manuscript, as indicated in the Author Contributions section. This involvement did not affect the objectivity of the work.

The updated Conflicts of Interest statement appears below:

**Conflicts of Interest:** Ł.R. and M.R. are co-founders of the Rodzen Brothers Foundation. K.B. (Karolina Bartoń) is the founder and owner of Karolina Barton Food Coach. J.U. is affiliated with the Collaborative Health Community Foundation. P.M. is the founder and CEO of Funmed Group, a functional medicine organization.

The authors state that the scientific conclusions are unaffected. This correction was approved by the Academic Editor. The original publication has also been updated.
